# Age determination for whitespotted conger *Conger myriaster* through somatic and otolith morphometrics

**DOI:** 10.1371/journal.pone.0203537

**Published:** 2018-09-05

**Authors:** Xiuxia Mu, Chongliang Zhang, Chi Zhang, Binduo Xu, Ying Xue, Yiping Ren

**Affiliations:** 1 College of Fisheries, Ocean University of China, Qingdao, Shandong, China; 2 Laboratory for Marine Science and Food Production Processes, National Laboratory for Marine Science and Technology, Qingdao, Shandong, China; Leibniz Institute on aging - Fritz Lipmann Institute (FLI), GERMANY

## Abstract

It is difficult to determine ages of eels via otoliths, because multiple alternating translucent and opaque zones in the otoliths are hard to identify. In this study, we developed an efficient age determination method for whitespotted conger (*Conger myriaster*), using random forest models with otolith weight and length, total body length, capture location and season as predictors. 409 specimens were collected from six locations in Yellow and East China Sea between October 2016 and December 2017. Overall OOB error rate was 17.36% compared with 16.26% for the external cross-validation dataset, and the error of age was within one year. Otolith weight and total length were the most important predictors, followed by otolith length, capture locations and seasons. There were no significant differences between the results derived from otolith/somatic morphometrics and otoliths annuli in the estimation of age composition and von Betalanffy Growth Functions growth curve. Our results demonstrated that random forest model with otolith and somatic morphometrics is an efficient and reliable approach for age determination of *C*. *myriaster*, which may also be applied to other eel species.

## Introduction

Eels are typically long-distance migratory fishes, widely distributed, and their life history is complex, spawning in warmer environmental conditions, drifting to colder waters as leptocephali, and undergoing metamorphosis [1]. Most eels are commercially important species, but the stock of those species have declined considerably in recent years, especially in East Asia (FAO, Global production statistics 1950–2015). Sound management plans become urgent for eels, however, management of eels using stock assessment methods is challenging, partially due to difficulty in age determination. That is, otolith is the most widely used material to estimate the age, and counts of growth increments in sectioned otoliths is considered as a reasonable approximation of age [2]. Unfortunately, there are usually multiple opaque zones with translucent zones in the otoliths of eels, making it difficult to read for elder eels [2–7].

Katayama introduced a new age method for the C. *myriaster* using UV light observation of burnt otoliths to make the annuli relatively easy to read [8], which is also applicable to other eel species, such as *Conger conger* [9] and *Anguilla rostrata* [10]. However, eels’ otoliths require more complex preparations prior to age determination, which is time-consuming. Besides, such methods need well-trained age readers, and the subjectivity of age reading is unavoidable. Therefore, an efficient and reliable age analysis method is needed for eel studies.

Despite the difficulties, otolith is a solid indicator of ages in other ways [11]. Studies suggested that the morphometrics of otoliths may provide critical information for aging when annuli is less useful. Radtke (1985) explored the utility of otolith weight to estimate ages of fish [12], and otolith morphometrics have been successful applied to determine age of eels [13–14]. In addition, age and total length (TL) are usually correlated, so somatic morphometrics could also be used for age determination [15–16].

Many statistical methods were used to avoid subjectivity in age determination using otolith/somatic morphometrics, such as random forests (RF) [16–18], maximum likelihood-based mixture analysis [15, 19], discriminant analysis [20], support vector machine [21], and ensemble of wrappers [22]. Random forest model is one of the most widely used approaches, which can model non-linear relationships and interactions [23]. This approach has been successfully applied to freshwater and long-lived marine species [16–18], providing a promising alternative to parametric approaches [17].

Although the random forest model is flexible but with limited reliability, because the algorithm may involve complex patterns between ages and predictors. Therefore, it is desirable to conduct systematic examinations on the predictive performance of the model. In this study, we evaluate the performance of random forest models using otolith and somatic morphometrics to estimate the age of a common eel species, whitespotted conger (*Conger myriaster*).

## Materials and methods

### Specimen collection and processing

**Fig 1:**
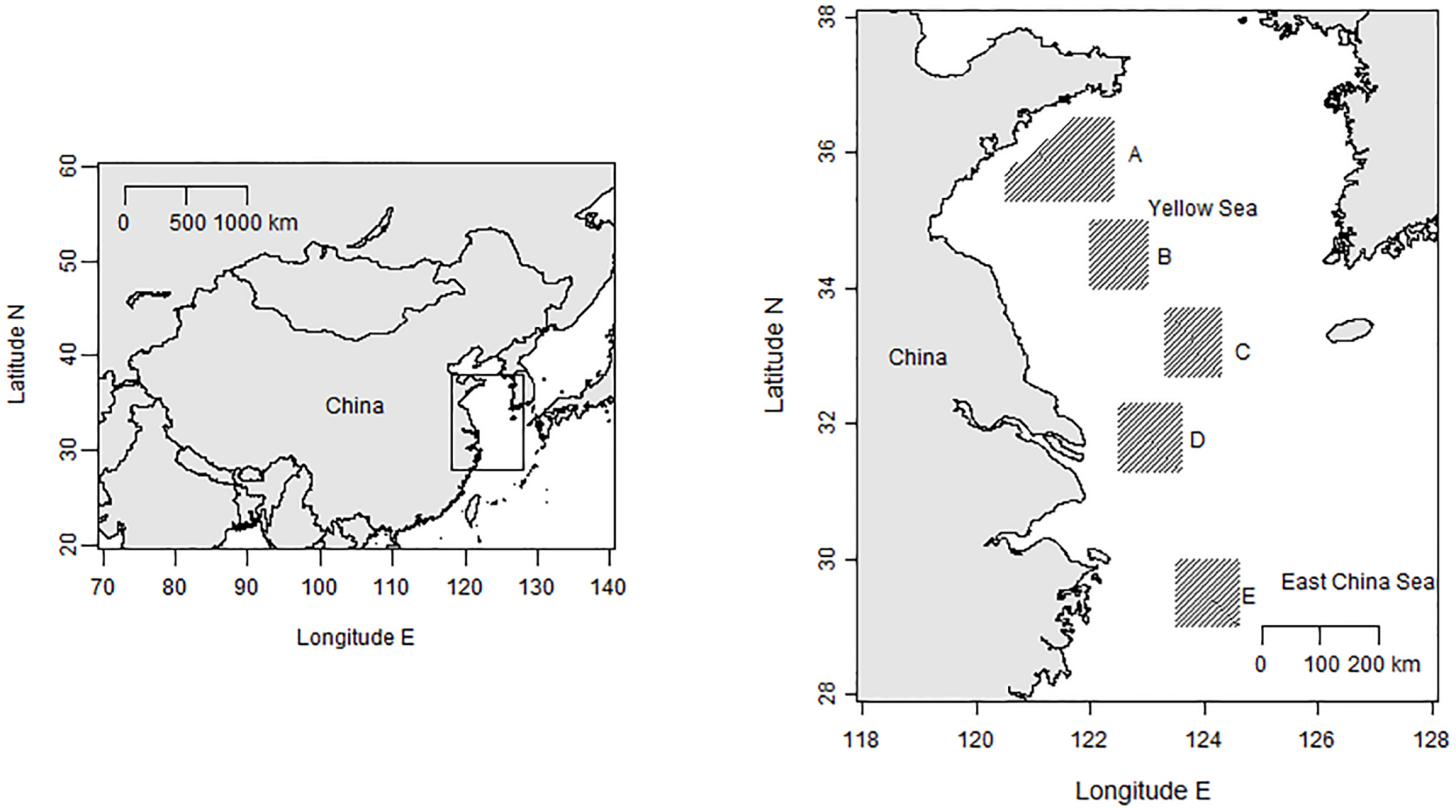
Survey areas for *Conger myriaster* in the Yellow and East China Seas. Rectangles indicate the approximate fishing areas of samples.

The conger specimens were collected using a bottom trawl in six survey areas located in the Yellow and East China Seas (Fig 1). A total of 409 specimens were used for age determination (Table 1). Samplings in area A were conducted using the trawl net of 15 m width and cod end mesh size of 20 mm, and samples from other areas were collected by local commercial bottom trawls.

**Table 1:** Sample size of *Conger myriaster* specimens collected in the Yellow and East China Seas.

survey season	survey area	survey time	number of specimens
spring	A	April and May, 2017	90
spring	B	April, 2017	24
spring	E	April, 2017	37
summer	A	August, 2017	45
summer	B	August, 2017	7
autumn	A	October, 2016; September, 2017	79
autumn	B	November, 2017	7
autumn	D	November, 2017	10
winter	A	January and December, 2017	74
winter	B	December, 2017	14
winter	C	February, 2017	9
winter	D	February, 2017	8
winter	E	December, 2017	5
total			409

See details for the survey area A, B, C, D, and E from Fig 1

**Fig 2:**
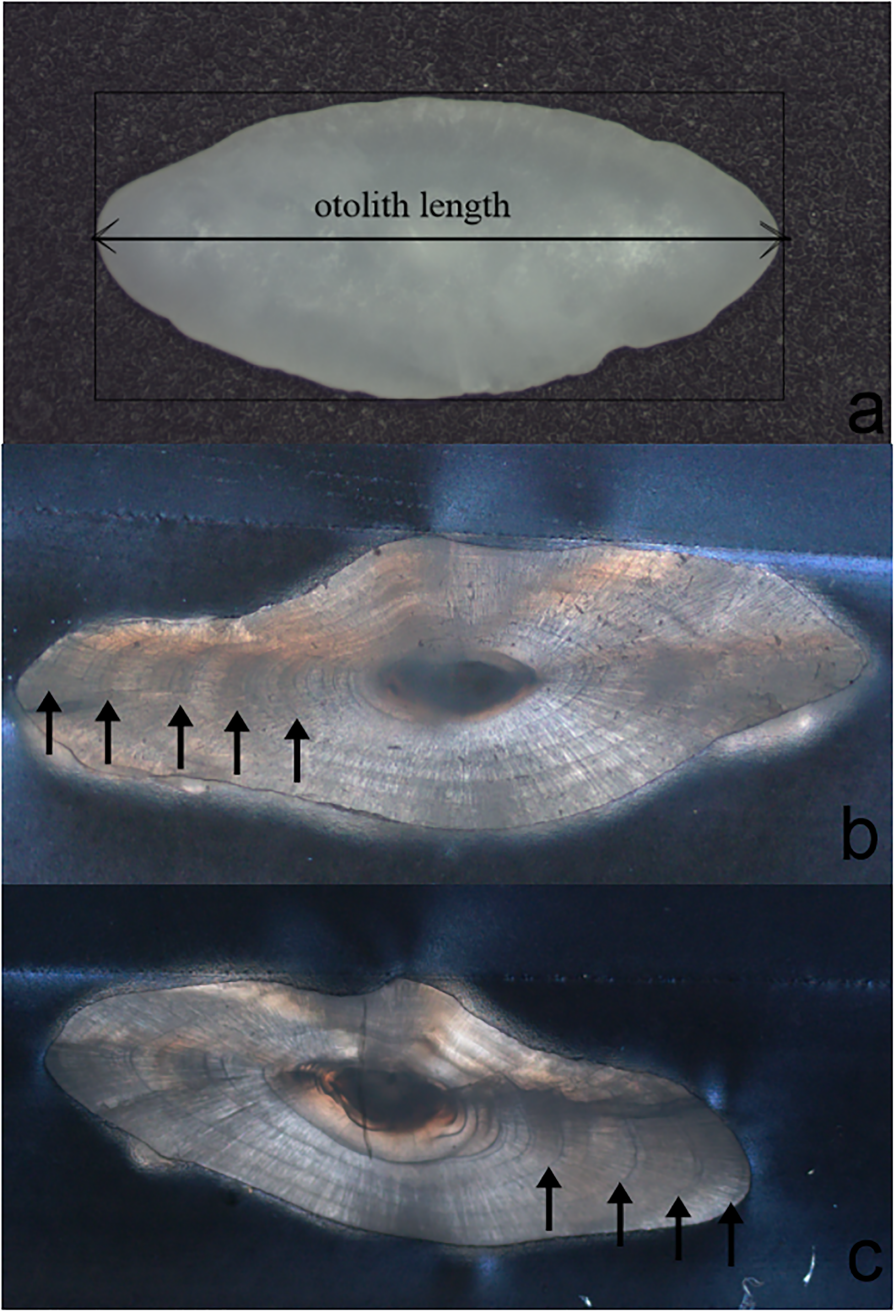
Digital photograph of sagittal otoliths (right otolith, convex outer side) of *Conger myriaster* showing measurements analyzed in this study(a) and the transverse sectioned otolith of *Conger myriaster* (b, c).

Total length (TL, mm) for each specimen was measured. Sagittal otoliths were dissected through the gills for age determination. Otoliths with chips or damage were not used in the analysis. Every right otolith from each fish were measured using an electronic balance (BP211D; Sartorius AG Gottingen). Otoliths were photographed on the convex outer surface using a Nikon SMZ800 at magnifications 2×. Otolith length (the horizontal distance between the anterior and the posterior tips of the otolith) was measured on the otolith images by software image J with an accuracy of 0.01 mm (Fig 2).

Each right sagittal otolith was heated at 200°C for 5–10 min, then embedded in epoxy resin (EpoThin, Buehler). The otoliths were then sectioned along the dorsal-ventral axis across the core with a diamond circular saw (Isomet low speed, Buehler). The sections (0.3 mm) were mounted on glass slides and polished with 800–1200 grit grinding paper (Minimet 1000 grinder-polisher, Buehler) until a clear pattern of any annuli was evident. Based on the assumption that C. *myriaster* hatches on 1 December [24], we estimated the age by counting annuli. All individual ages are presented as whole numbers representing complete annuli (Fig 2). Ages were randomly read twice at a half month interval without fish information, and the conflicting results were determined by a well-experienced reader with fish information, our results were assumed independent age estimates without known aged fishes.

### Statistical analyses

Random forest models were used to estimate the age of C. *myriaster* from otolith and somatic morphometric data using the random forest package (version V 3.1) [25] in R. The random forest method is an improvement over the standard bagged tree approach. This algorithm firstly selects many bootstrap samples from the data, each of which contains 63% of the original observations [23]. The others are referred to as OOB (out-of-bag) observations used to estimate misclassification rate and serve as a form of cross-validation when external test samples are not available [23]. Trees are fitted to bootstrap samples without pruning, and the trees are combined to predict the OOB observations. The predicted result is calculated by averaging the OOB predicted values across all trees. Importance of each predictor variable are assessed by comparing the mean decrease in accuracy.

Age was predicted using otolith weight (OW) and otolith length (OL). In addition, TL, locations of capture (sample area) and seasons were also included in the model to account for the spatio-temporal variation of ages. A large number of trees (2,000) were used in our model, and different random seeds were applied to ensure model robustness. We trained models with different mtry value and identified the optimal value mtry = 2 when RF performed best. We reported and compared both the OOB error rate and error rate obtained when the resulting forest was applied to predict the ages of C. *myriaster* from a separate test dataset for cross-validation. In cross-validation, the original data set was randomly partitioned into train set (70% samples), which was used to fit the model; and test set (30% samples), which was used to evaluate the predictive performance. This process was repeated for 1000 times, and we calculated the mean error rate for the external test dataset.

Two approaches were used to compare model-predicted ages with ages estimated by annual ring counts from the test dataset: (i) chi-square test was used to examine the differences of age frequency distribution between model-based and counted ages, (ii) von-Bertalanffy growth models (VBGF) were fitted and an analysis of the residual sum of squares (ARSS) was employed to compare VBGF between the counted age and the model-based age.[26]. Growth was presented by the von-Bertalanffy growth equation[27]:

$$L_{t}=L_{\infty }(1-e^{-k(t-t_{0})})$$

Where *L*_*t*_ is the total length at age t, *L*_∞_ is the asymptotic length, k is the growth coefficient, and t_0_ is the hypothetical age at zero length. Growth model was implemented using the packages FSA in R 3.3.3 [28].

## Results

**Fig 3:**
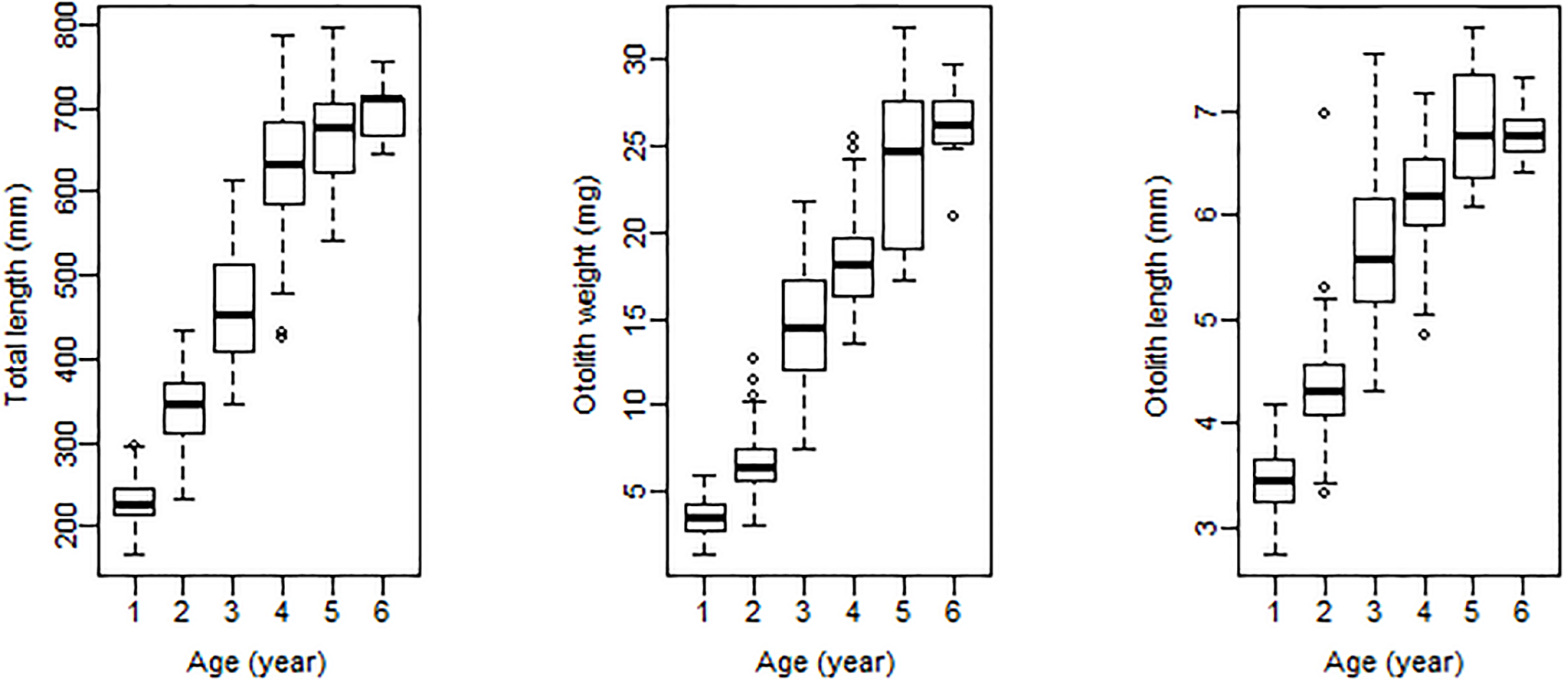
The distribution of fish total lengths, otolith weights and otolith length observed at each age (years) for *Conger myriaster* (n = 409).

**Fig 4:**
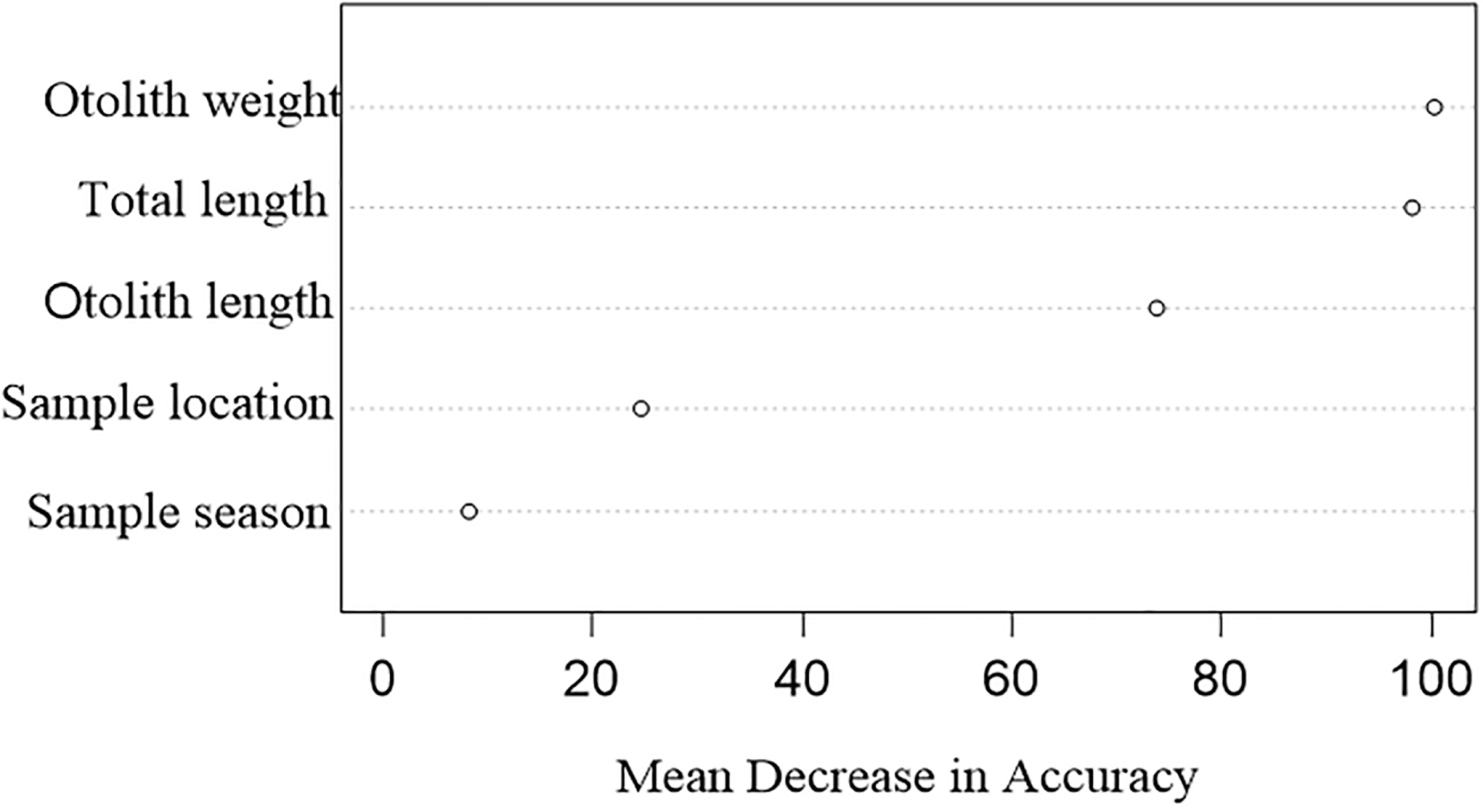
Variable importance indices for classifying *Conger myriaster* ages using the random forest model.

The minimum and maximum observed ages of C. *myriaster* were one and six years, respectively. The TL ranged from 165 to 795 mm, and the otolith weight and length ranged from 1.3 to 31.8 mg and 2.7 to 7.8 mm, respectively (Fig 3). Overall OOB error rate was 17.36%, compared with 16.26% error for the external cross-validation dataset (Table 2). The predicted age derived from otolith and somatic morphologic data also ranged from one to six years old, and the differences of age of misclassified fish was no more than 1 years (Table 2). No significant differences were found in the age frequency distribution between model-based and counted ages for C. *myriaster* (Table 2, *x*^*2*^ = 0.36, d. f. = 5, *p* = 0.996). The otolith weight was the most important predictor for age, followed by total length, otolith length and sample locations, whereas season was the least important (Fig 4).

**Table 2:** Counted age versus predicted age of *Conger myriaster* in the test dataset sampled from October 2016 to December 2017.

		**model-based age**					
		**age1**	**age2**	**age3**	**age4**	**age5**	**Age6**
**counted age**	**age1**	0.89	0.11	0	0	0	0
	**age2**	0.06	0.9	0.04	0	0	0
	**age3**	0	0.125	0.75	0.125	0	0
	**age4**	0	0	0.11	0.72	0.17	0
	**age5**	0	0	0	0.44	0.44	0.12
	**age6**	0	0	0	0	1	0

The values represent the probability of each fish classified in to different age groups

**Fig 5:**
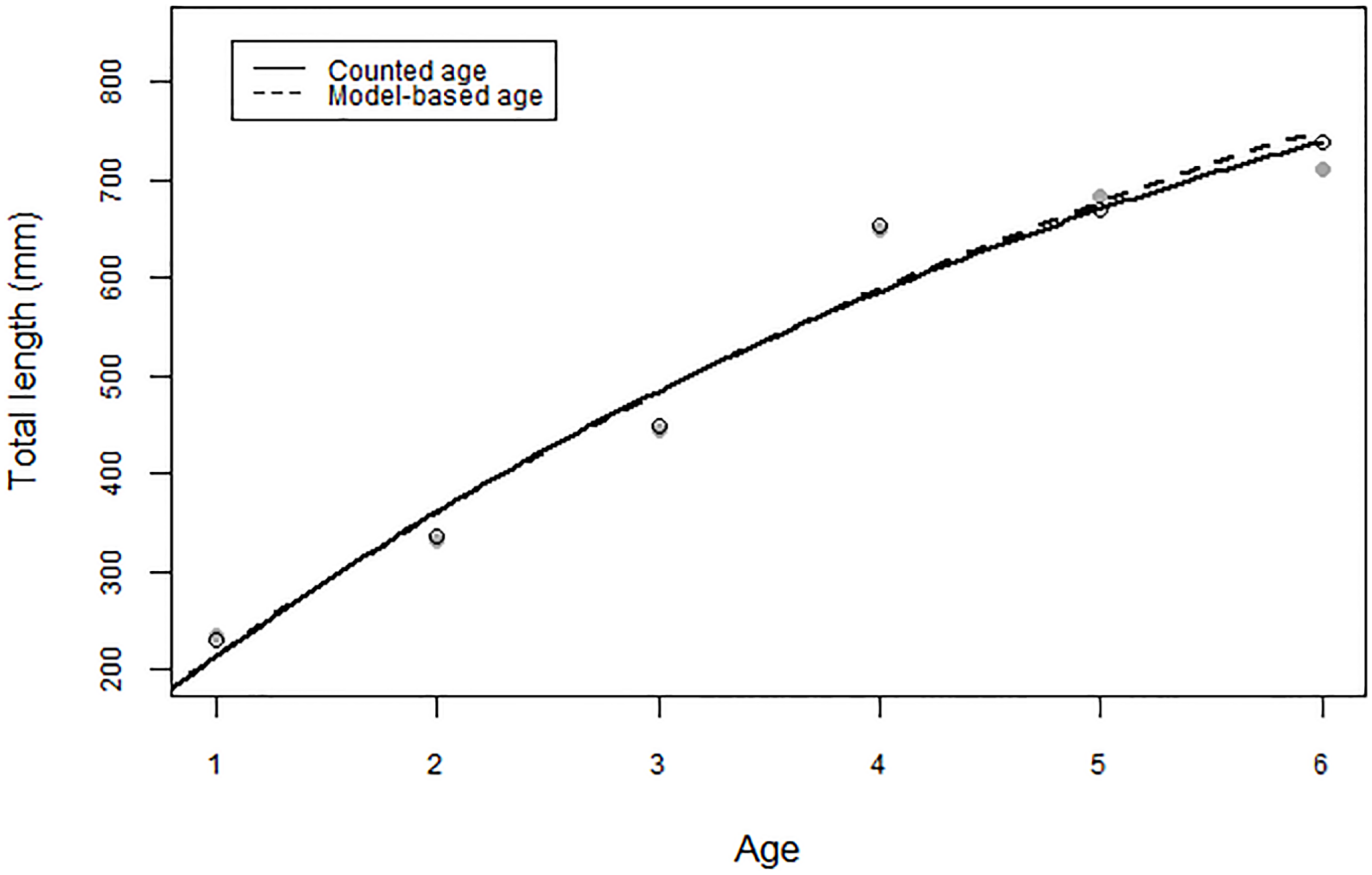
Von Bertalanffy growth curves for mean length at age for model-based age (dashed line) and counted age (solid line). Open circles represent the mean total length at every counted age class, and the solid circles represent the mean total length at every model-based age class.

The population growth curves fitted using counted age and model-based age were similar (Fig 5). The mean total length at counted age was described by the von Bertalanffy equation, L_t_ = 1073 × (1 –e^−0.189×(t+0.168)^), and the mean total length at model-based age was described by the von Bertalanffy equation, L_t_ = 1165 × (1 –e^−0.1665×(t+0.223)^). ARSS on the VBGF indicted that no differences in VBGF between the counted age and model-based age (F = 1.009, *p* = 0.433).

## Discussion

We demonstrated that aging methods using otolith and somatic morphometric data can estimate age composition and growth as accurately as those using otolith increment. Error rates of the model-based age was acceptable for many standard fisheries assessments [29]. Prediction error of the OOB sample was slightly greater than the test dataset error, suggesting that the train samples had sufficient range to accurately predict the ages of the test samples. The RF model performed well at predicting the age of the fish for ages 1–4 but have some limitation for older ones. Considering that the samples collected from coastal China seas were all young individuals, we think the model could also provide valuable information in the coastal seas despite the limitation. On the other hands, caveats should be taken when large individuals were involved in this model.

Using somatic/otolith size or shapes is usually questionable for age estimation, because the growth of fish tended to slow down when getting older, so that many morphometric measures overlapped among elder age groups. For examples, body length was a poor age predictor for long-lived fish, such as *Perca flavescens* and deep-water snappers [18]. However, this problem is less important for short-lived and fast-growing fishes, as shown in the age determination for *Oncorhynchus nerka* [17]. Similarly, the importance of total length in predicting the ages of C. *myriaster* may be attributed to small size of eels captured in our study. Eels collected from coastal China seas were all young individuals up to 6 years, and older fish may prefer offshore areas. Otolith weight is more reliable to predict age compared with other morphological characteristics of otolith or body [17–19], which could be attributed to the fact that the growth of otolith and body length of C. *myriaster* are not synchronized [24, 30]. The otolith weight increases continually over the life, while the growth of body and otolith length slows down with ages [11, 31]. In addition, the total length of leptocephali diminishes during metamorphosis, but the otolith continues to grow [24].

The capture location is an important predictor of ages, which may be due to the age-dependent distribution of C. *myriaster*. Previous studies found that juvenile C. *myriaster* mainly distributed in the coastal waters [32–33], and then migrated southward to continental shelf of East China Sea with growth [34]. Therefore, the ages of fish would be different among capture locations due to different habitat preference. Besides, specimens were collected over a wide spatial range and might not come from the same stock.

Age information are commonly fit to growth models to get catch-at-age data in stock assessments [16]. Our study suggested that the fitted growth models using model-based age and counted age were not significantly different, implying that otolith annuli analyses may not be necessary to growth analyses, instead, otolith and fish morphometrics can be used. Besides, the *L*_∞_ and K estimated in the present study are similar to those reported in the Southern water of Korea [14, 35], supporting the usability of this approach.

Our results demonstrate that otolith and somatic morphometrics provide useful information to estimate age and growth pattern of C. *myriaster* and the RF model is efficient in age determination. This approach is important to age determination of eels whose age determination is difficult. In addition, this approach can greatly simplify the process needed to age determination, especially for estimation growth rate. Whereas, the application of this approach should be cautious when collecting samples across large areas and choosing suitable somatic morphometrics.

## Supporting information

S1 Table Otolith and somatic morphometric data of *Conger myriaster* used in the random forest model. (DOCX)
