# Supplementary material for: Age determination for whitespotted conger Conger myriaster through somatic and otolith morphometrics
Source: PLoS One. 2018 Sep 5;13(9):e0203537. doi: 10.1371/journal.pone.0203537 (PMC6124768; doi:10.1371/journal.pone.0203537)
Supplement: S1 Table — (DOCX) [file pone.0203537.s001.docx]

| S Table. Otolith and somatic morphometric data of *Conger myriaster* used in the random forest model. | | | | | | | |
| --- | --- | --- | --- | --- | --- | --- | --- |
| number | sample location | sample season | sample time | otolith weight/mg | counted age/year | total length/mm | otolith /mm |
| 1 | A | spring | May, 2017 | 5.87 | 2 | 239 | 4.27 |
| 2 | A | spring | May, 2017 | 6.1 | 2 | 283 | 3.95 |
| 3 | A | summer | August, 2017 | 6.14 | 2 | 291 | 4.36 |
| 4 | A | winter | January, 2017 | 6.04 | 2 | 309 | 4.30 |
| 5 | A | autumn | October, 2016 | 6.21 | 2 | 354 | 4.52 |
| 6 | A | autumn | September, 2017 | 8.3 | 2 | 318 | 4.58 |
| 7 | A | spring | May, 2017 | 4.39 | 2 | 240 | 3.31 |
| 8 | A | winter | January, 2017 | 3.08 | 2 | 241 | 3.42 |
| 9 | A | autumn | September, 2017 | 4.24 | 2 | 435 | 3.48 |
| 10 | A | spring | April, 2017 | 5.7 | 2 | 313 | 3.52 |
| 11 | A | spring | May, 2017 | 4.01 | 2 | 233 | 3.55 |
| 12 | A | winter | January, 2017 | 6.44 | 2 | 320 | 4.74 |
| 13 | A | winter | December, 2017 | 4.5 | 2 | 257 | 3.71 |
| 14 | A | summer | August, 2017 | 2.29 | 1 | 165 | 2.89 |
| 15 | A | summer | August, 2017 | 2.26 | 1 | 176 | 2.92 |
| 16 | A | autumn | September, 2017 | 2.2 | 1 | 176 | 2.90 |
| 17 | A | spring | May, 2017 | 4.1 | 2 | 251 | 3.74 |
| 18 | A | autumn | September, 2017 | 2.23 | 1 | 178 | 2.90 |
| 19 | A | spring | April, 2017 | 6.1 | 2 | 329 | 3.75 |
| 20 | A | autumn | October, 2016 | 1.3 | 1 | 184 | 2.89 |
| 21 | A | spring | April, 2017 | 4.6 | 2 | 297 | 3.80 |
| 22 | A | winter | January, 2017 | 3.23 | 2 | 250 | 3.80 |
| 23 | A | spring | May, 2017 | 3.06 | 1 | 185 | 3.00 |
| 24 | B | spring | April, 2017 | 15.89 | 4 | 426 | 5.39 |
| 25 | A | autumn | October, 2016 | 5.29 | 2 | 325 | 4.16 |
| 26 | A | spring | May, 2017 | 6.86 | 2 | 373 | 4.89 |
| 27 | A | winter | January, 2017 | 7.19 | 2 | 395 | 5.09 |
| 28 | A | autumn | October, 2016 | 3.54 | 2 | 268 | 3.84 |
| 29 | A | winter | January, 2017 | 7.26 | 2 | 375 | 4.79 |
| 30 | A | spring | April, 2017 | 6.5 | 2 | 365 | 3.84 |
| 31 | A | spring | May, 2017 | 3.55 | 1 | 190 | 3.43 |
| 32 | A | spring | May, 2017 | 10.59 | 2 | 341 | 4.48 |
| 33 | A | spring | May, 2017 | 7.86 | 2 | 345 | 4.39 |
| 34 | A | autumn | September, 2017 | 8.6 | 2 | 344 | 4.65 |
| 35 | A | spring | May, 2017 | 8.41 | 2 | 351 | 4.54 |
| 36 | A | spring | May, 2017 | 10.2 | 2 | 344 | 4.95 |
| 37 | A | spring | May, 2017 | 13.53 | 3 | 345 | 5.20 |
| 38 | A | spring | May, 2017 | 7.95 | 2 | 326 | 4.35 |
| 39 | A | spring | May, 2017 | 9.01 | 2 | 351 | 4.84 |
| 40 | A | summer | August, 2017 | 6.2 | 2 | 362 | 4.26 |
| 41 | B | summer | August, 2017 | 16.47 | 4 | 430 | 5.63 |
| 42 | A | autumn | October, 2016 | 6.27 | 2 | 362 | 4.21 |
| 43 | A | spring | May, 2017 | 3.3 | 1 | 190 | 3.13 |
| 44 | B | summer | August, 2017 | 16.95 | 4 | 506 | 5.94 |
| 45 | A | winter | January, 2017 | 6.21 | 2 | 363 | 4.58 |
| 46 | A | summer | August, 2017 | 7.96 | 2 | 365 | 4.36 |
| 47 | A | autumn | October, 2016 | 8.25 | 2 | 394 | 4.77 |
| 48 | A | summer | August, 2017 | 2.61 | 1 | 194 | 3.07 |
| 49 | A | winter | January, 2017 | 1.99 | 1 | 194 | 3.10 |
| 50 | A | spring | May, 2017 | 10.15 | 2 | 352 | 4.49 |
| 51 | A | spring | April, 2017 | 5.6 | 2 | 357 | 3.86 |
| 52 | A | winter | December, 2017 | 2.52 | 1 | 195 | 3.03 |
| 53 | A | winter | December, 2017 | 5.4 | 2 | 314 | 3.88 |
| 54 | A | autumn | September, 2017 | 2.9 | 1 | 196 | 3.17 |
| 55 | D | autumn | November, 2017 | 6.3 | 2 | 367 | 4.10 |
| 56 | A | spring | May, 2017 | 8.36 | 2 | 361 | 4.73 |
| 57 | A | summer | August, 2017 | 12.48 | 3 | 375 | 5.93 |
| 58 | A | autumn | September, 2017 | 2.91 | 1 | 200 | 3.07 |
| 59 | A | spring | April, 2017 | 5.9 | 2 | 369 | 3.89 |
| 60 | A | spring | April, 2017 | 6.1 | 2 | 336 | 3.91 |
| 61 | A | spring | April, 2017 | 5.7 | 2 | 311 | 3.92 |
| 62 | A | autumn | September, 2017 | 3.6 | 1 | 203 | 3.60 |
| 63 | A | winter | January, 2017 | 2.06 | 1 | 203 | 3.27 |
| 64 | A | spring | April, 2017 | 6.2 | 2 | 312 | 3.92 |
| 65 | A | winter | January, 2017 | 1.89 | 1 | 204 | 3.06 |
| 66 | A | winter | January, 2017 | 5.33 | 2 | 382 | 4.02 |
| 67 | A | winter | December, 2017 | 3.7 | 1 | 208 | 3.46 |
| 68 | A | autumn | September, 2017 | 2.93 | 1 | 208 | 3.16 |
| 69 | A | spring | May, 2017 | 12.5 | 3 | 378 | 5.16 |
| 70 | A | winter | January, 2017 | 7.21 | 2 | 384 | 4.57 |
| 71 | A | autumn | October, 2016 | 4.21 | 2 | 365 | 3.92 |
| 72 | A | summer | August, 2017 | 6.8 | 2 | 386 | 4.29 |
| 73 | A | autumn | September, 2017 | 5.6 | 2 | 270 | 3.93 |
| 74 | A | autumn | September, 2017 | 3.5 | 1 | 210 | 3.36 |
| 75 | A | spring | May, 2017 | 11.48 | 3 | 383 | 5.29 |
| 76 | A | autumn | September, 2017 | 2.9 | 1 | 210 | 3.34 |
| 77 | A | winter | January, 2017 | 6.46 | 2 | 388 | 4.60 |
| 78 | D | autumn | November, 2017 | 7.37 | 2 | 385 | 4.41 |
| 79 | A | winter | January, 2017 | 9.39 | 2 | 310 | 4.80 |
| 80 | A | autumn | October, 2016 | 8.31 | 2 | 390 | 4.90 |
| 81 | A | spring | April, 2017 | 6.4 | 2 | 319 | 3.94 |
| 82 | A | autumn | October, 2016 | 5.58 | 2 | 395 | 4.55 |
| 83 | A | winter | December, 2017 | 8.51 | 3 | 390 | 4.93 |
| 84 | B | winter | December, 2017 | 7.41 | 3 | 397 | 4.31 |
| 85 | A | spring | April, 2017 | 5.6 | 2 | 315 | 3.97 |
| 86 | A | winter | January, 2017 | 5.47 | 2 | 347 | 3.97 |
| 87 | A | winter | January, 2017 | 8.02 | 3 | 398 | 4.82 |
| 88 | A | spring | May, 2017 | 9.74 | 2 | 399 | 4.56 |
| 89 | B | spring | April, 2017 | 7.5 | 2 | 401 | 6.97 |
| 90 | B | spring | April, 2017 | 9.9 | 3 | 401 | 6.72 |
| 91 | A | spring | May, 2017 | 9.93 | 2 | 401 | 4.71 |
| 92 | A | winter | December, 2017 | 5.04 | 2 | 260 | 3.98 |
| 93 | A | spring | April, 2017 | 6.1 | 2 | 372 | 3.98 |
| 94 | A | summer | August, 2017 | 11.16 | 3 | 405 | 5.23 |
| 95 | B | summer | August, 2017 | 15.24 | 4 | 525 | 5.04 |
| 96 | E | spring | April, 2017 | 18.1 | 4 | 533 | 6.54 |
| 97 | A | spring | May, 2017 | 11.66 | 3 | 406 | 5.37 |
| 98 | A | summer | August, 2017 | 5.62 | 2 | 285 | 3.98 |
| 99 | A | autumn | September, 2017 | 12.4 | 3 | 409 | 5.27 |
| 100 | A | spring | May, 2017 | 13.01 | 3 | 409 | 4.98 |
| 101 | A | spring | May, 2017 | 13.09 | 3 | 409 | 5.19 |
| 102 | A | summer | August, 2017 | 11.69 | 3 | 410 | 4.50 |
| 103 | A | winter | December, 2017 | 5 | 2 | 253 | 4.01 |
| 104 | A | autumn | September, 2017 | 5.97 | 2 | 302 | 4.02 |
| 105 | A | spring | April, 2017 | 6.2 | 2 | 321 | 4.02 |
| 106 | A | spring | May, 2017 | 14.27 | 3 | 410 | 4.99 |
| 107 | B | spring | April, 2017 | 25.4 | 4 | 549 | 6.45 |
| 108 | A | spring | April, 2017 | 5.8 | 2 | 355 | 4.02 |
| 109 | B | winter | December, 2017 | 19.73 | 4 | 560 | 6.38 |
| 110 | A | winter | January, 2017 | 2.58 | 1 | 210 | 3.66 |
| 111 | A | autumn | September, 2017 | 3.06 | 1 | 212 | 3.30 |
| 112 | A | spring | May, 2017 | 14.95 | 3 | 410 | 5.57 |
| 113 | A | summer | August, 2017 | 9.47 | 3 | 413 | 4.30 |
| 114 | A | spring | May, 2017 | 13.41 | 3 | 415 | 5.09 |
| 115 | A | summer | August, 2017 | 5.36 | 2 | 289 | 4.03 |
| 116 | A | spring | May, 2017 | 16.41 | 3 | 415 | 5.43 |
| 117 | B | winter | December, 2017 | 7.41 | 3 | 425 | 4.56 |
| 118 | A | spring | May, 2017 | 12.72 | 2 | 425 | 5.20 |
| 119 | B | spring | April, 2017 | 21.1 | 3 | 432 | 6.27 |
| 120 | A | summer | August, 2017 | 11.44 | 2 | 433 | 5.29 |
| 121 | A | autumn | September, 2017 | 13.8 | 3 | 435 | 5.09 |
| 122 | A | spring | May, 2017 | 16.26 | 3 | 437 | 5.53 |
| 123 | B | winter | December, 2017 | 9.2 | 2 | 432 | 4.81 |
| 124 | A | winter | December, 2017 | 8.63 | 3 | 440 | 4.51 |
| 125 | A | summer | August, 2017 | 16.81 | 3 | 441 | 5.40 |
| 126 | A | summer | August, 2017 | 12.05 | 3 | 443 | 5.63 |
| 127 | A | spring | May, 2017 | 15.4 | 3 | 443 | 5.00 |
| 128 | A | summer | August, 2017 | 15.49 | 3 | 453 | 5.56 |
| 129 | B | autumn | November, 2017 | 24.3 | 4 | 565 | 6.17 |
| 130 | A | spring | May, 2017 | 14.46 | 3 | 454 | 5.29 |
| 131 | B | winter | December, 2017 | 18.79 | 4 | 567 | 5.62 |
| 132 | A | spring | April, 2017 | 5.3 | 2 | 322 | 4.04 |
| 133 | A | spring | May, 2017 | 16.38 | 3 | 456 | 5.58 |
| 134 | B | spring | April, 2017 | 17.4 | 3 | 462 | 7.19 |
| 135 | B | spring | April, 2017 | 15.5 | 3 | 463 | 6.43 |
| 136 | A | spring | May, 2017 | 17.27 | 3 | 467 | 5.96 |
| 137 | B | autumn | November, 2017 | 19 | 4 | 572 | 5.95 |
| 138 | B | summer | August, 2017 | 22.44 | 4 | 573 | 6.63 |
| 139 | B | winter | December, 2017 | 16.77 | 3 | 468 | 6.75 |
| 140 | B | winter | December, 2017 | 18.57 | 4 | 585 | 6.02 |
| 141 | D | autumn | November, 2017 | 19.72 | 4 | 587 | 6.66 |
| 142 | E | spring | April, 2017 | 16.5 | 4 | 592 | 5.87 |
| 143 | A | summer | August, 2017 | 11.79 | 3 | 469 | 4.90 |
| 144 | B | summer | August, 2017 | 15.82 | 4 | 602 | 4.83 |
| 145 | A | spring | May, 2017 | 21.25 | 3 | 470 | 5.89 |
| 146 | B | spring | April, 2017 | 16.91 | 4 | 479 | 5.39 |
| 147 | A | winter | December, 2017 | 5.44 | 2 | 340 | 4.04 |
| 148 | B | summer | August, 2017 | 14.75 | 4 | 480 | 5.63 |
| 149 | B | spring | April, 2017 | 17.76 | 4 | 484 | 5.95 |
| 150 | A | spring | April, 2017 | 6.4 | 2 | 314 | 4.04 |
| 151 | A | summer | August, 2017 | 2.59 | 1 | 212 | 3.01 |
| 152 | A | autumn | October, 2016 | 5.07 | 2 | 348 | 4.04 |
| 153 | B | summer | August, 2017 | 20.42 | 3 | 485 | 5.79 |
| 154 | A | winter | December, 2017 | 5.46 | 2 | 285 | 4.05 |
| 155 | A | summer | August, 2017 | 14.42 | 3 | 487 | 5.93 |
| 156 | A | winter | January, 2017 | 2.43 | 1 | 213 | 3.44 |
| 157 | A | autumn | September, 2017 | 3.4 | 1 | 214 | 3.09 |
| 158 | A | winter | January, 2017 | 2.28 | 1 | 214 | 3.51 |
| 159 | B | spring | April, 2017 | 21.8 | 3 | 489 | 6.27 |
| 160 | A | summer | August, 2017 | 3.93 | 1 | 215 | 3.25 |
| 161 | A | spring | April, 2017 | 6.6 | 2 | 345 | 4.05 |
| 162 | A | summer | August, 2017 | 5.92 | 2 | 347 | 4.07 |
| 163 | A | spring | April, 2017 | 5.6 | 2 | 339 | 4.07 |
| 164 | A | spring | May, 2017 | 5.75 | 2 | 386 | 4.07 |
| 165 | A | autumn | October, 2016 | 4.69 | 2 | 289 | 4.07 |
| 166 | A | summer | August, 2017 | 6.06 | 2 | 338 | 4.09 |
| 167 | A | autumn | October, 2016 | 4.89 | 2 | 359 | 4.09 |
| 168 | A | winter | January, 2017 | 2.87 | 1 | 215 | 3.26 |
| 169 | E | winter | December, 2017 | 16.37 | 4 | 610 | 6.25 |
| 170 | A | autumn | September, 2017 | 3.17 | 1 | 217 | 3.30 |
| 171 | A | winter | January, 2017 | 2.63 | 1 | 217 | 3.28 |
| 172 | A | winter | January, 2017 | 2.52 | 1 | 217 | 3.30 |
| 173 | A | autumn | September, 2017 | 3.24 | 1 | 218 | 3.23 |
| 174 | A | spring | May, 2017 | 3.44 | 1 | 219 | 3.53 |
| 175 | A | winter | January, 2017 | 2.46 | 1 | 219 | 3.28 |
| 176 | A | autumn | October, 2016 | 2.13 | 1 | 219 | 3.34 |
| 177 | A | autumn | September, 2017 | 3.5 | 1 | 222 | 3.21 |
| 178 | A | summer | August, 2017 | 2.98 | 1 | 222 | 3.17 |
| 179 | A | autumn | September, 2017 | 2.9 | 1 | 222 | 3.30 |
| 180 | A | winter | December, 2017 | 3.8 | 1 | 224 | 3.46 |
| 181 | A | winter | December, 2017 | 3.42 | 1 | 224 | 3.22 |
| 182 | A | winter | January, 2017 | 2.49 | 1 | 224 | 3.28 |
| 183 | A | autumn | October, 2016 | 5.04 | 2 | 310 | 4.10 |
| 184 | A | summer | August, 2017 | 3.12 | 1 | 225 | 3.42 |
| 185 | A | winter | December, 2017 | 4.92 | 2 | 263 | 4.11 |
| 186 | A | winter | January, 2017 | 2.83 | 1 | 225 | 3.41 |
| 187 | A | summer | August, 2017 | 2.44 | 1 | 225 | 3.17 |
| 188 | A | spring | April, 2017 | 5.4 | 2 | 303 | 4.11 |
| 189 | A | summer | August, 2017 | 15.52 | 3 | 502 | 5.56 |
| 190 | A | summer | August, 2017 | 5.95 | 2 | 316 | 4.12 |
| 191 | A | summer | August, 2017 | 21.53 | 3 | 503 | 6.16 |
| 192 | A | autumn | September, 2017 | 10.32 | 3 | 514 | 5.10 |
| 193 | A | spring | April, 2017 | 6.3 | 2 | 351 | 4.12 |
| 194 | B | spring | April, 2017 | 17.9 | 3 | 514 | 7.56 |
| 195 | C | winter | February,2017 | 19.57 | 4 | 610 | 6.70 |
| 196 | A | spring | April, 2017 | 6 | 2 | 338 | 4.12 |
| 197 | A | spring | May, 2017 | 3.67 | 1 | 226 | 3.36 |
| 198 | A | summer | August, 2017 | 12.98 | 3 | 532 | 5.73 |
| 199 | A | summer | August, 2017 | 5.73 | 2 | 368 | 4.14 |
| 200 | A | autumn | September, 2017 | 17.2 | 3 | 535 | 5.78 |
| 201 | B | spring | April, 2017 | 18.45 | 3 | 538 | 5.65 |
| 202 | B | winter | December, 2017 | 18.79 | 4 | 615 | 6.07 |
| 203 | E | winter | December, 2017 | 18.48 | 4 | 619 | 6.42 |
| 204 | B | spring | April, 2017 | 18.8 | 3 | 544 | 6.70 |
| 205 | C | winter | February,2017 | 18.19 | 4 | 624 | 5.98 |
| 206 | B | spring | April, 2017 | 15.3 | 3 | 546 | 6.57 |
| 207 | A | summer | August, 2017 | 14.31 | 3 | 552 | 5.95 |
| 208 | B | winter | December, 2017 | 12.08 | 3 | 558 | 5.53 |
| 209 | E | spring | April, 2017 | 12.9 | 3 | 560 | 5.54 |
| 210 | B | autumn | November, 2017 | 22.72 | 4 | 626 | 6.44 |
| 211 | D | autumn | November, 2017 | 24.84 | 4 | 627 | 7.10 |
| 212 | B | spring | April, 2017 | 27.51 | 5 | 541 | 6.69 |
| 213 | E | winter | December, 2017 | 17.22 | 3 | 570 | 6.54 |
| 214 | E | spring | April, 2017 | 13.5 | 4 | 630 | 5.90 |
| 215 | D | winter | February,2017 | 18.1 | 4 | 631 | 6.83 |
| 216 | A | autumn | September, 2017 | 3.5 | 1 | 227 | 3.40 |
| 217 | D | winter | November,2017 | 24.84 | 5 | 557 | 6.15 |
| 218 | B | spring | April, 2017 | 17.1 | 3 | 577 | 6.38 |
| 219 | E | spring | April, 2017 | 22.02 | 4 | 632 | 6.63 |
| 220 | B | spring | April, 2017 | 18.7 | 3 | 580 | 6.48 |
| 221 | E | spring | April, 2017 | 16.28 | 4 | 634 | 5.92 |
| 222 | D | winter | February,2017 | 21.09 | 4 | 634 | 6.46 |
| 223 | E | spring | April, 2017 | 17.9 | 4 | 643 | 6.62 |
| 224 | D | autumn | November, 2017 | 20.44 | 5 | 573 | 6.94 |
| 225 | E | spring | April, 2017 | 15.9 | 4 | 644 | 6.00 |
| 226 | B | autumn | November, 2017 | 15.59 | 4 | 645 | 6.16 |
| 227 | B | spring | April, 2017 | 27.1 | 5 | 574 | 6.96 |
| 228 | E | spring | April, 2017 | 16.8 | 4 | 645 | 6.31 |
| 229 | E | winter | December, 2017 | 17.81 | 3 | 599 | 6.27 |
| 230 | E | spring | April, 2017 | 17.7 | 4 | 646 | 6.08 |
| 231 | E | spring | April, 2017 | 15.7 | 3 | 605 | 6.12 |
| 232 | E | spring | April, 2017 | 16.4 | 4 | 653 | 6.25 |
| 233 | E | spring | April, 2017 | 20.63 | 4 | 657 | 6.68 |
| 234 | B | winter | December, 2017 | 18.48 | 4 | 658 | 6.15 |
| 235 | E | spring | April, 2017 | 21.3 | 4 | 671 | 6.63 |
| 236 | D | winter | February,2017 | 22.76 | 4 | 674 | 6.38 |
| 237 | E | spring | April, 2017 | 16.7 | 4 | 678 | 5.86 |
| 238 | E | spring | April, 2017 | 19 | 4 | 678 | 6.12 |
| 239 | D | winter | February,2017 | 21.72 | 4 | 683 | 6.64 |
| 240 | D | winter | February,2017 | 18.39 | 4 | 687 | 6.21 |
| 241 | E | spring | April, 2017 | 20.93 | 4 | 689 | 6.61 |
| 242 | E | spring | April, 2017 | 18.6 | 4 | 691 | 6.30 |
| 243 | E | spring | April, 2017 | 19.4 | 4 | 692 | 6.68 |
| 244 | D | winter | February,2017 | 16.2 | 4 | 698 | 5.48 |
| 245 | B | spring | April, 2017 | 21.2 | 5 | 579 | 6.55 |
| 246 | E | spring | April, 2017 | 16.5 | 4 | 702 | 6.42 |
| 247 | B | winter | December, 2017 | 19.69 | 5 | 597 | 6.34 |
| 248 | E | spring | April, 2017 | 18.2 | 4 | 711 | 7.15 |
| 249 | B | autumn | November, 2017 | 22.55 | 5 | 618 | 6.35 |
| 250 | E | spring | April, 2017 | 15.3 | 4 | 720 | 6.22 |
| 251 | C | winter | February,2017 | 29.72 | 5 | 624 | 7.35 |
| 252 | D | winter | February,2017 | 21.17 | 5 | 638 | 6.28 |
| 253 | E | spring | April, 2017 | 15.6 | 4 | 723 | 5.69 |
| 254 | A | autumn | September, 2017 | 7.3 | 2 | 375 | 4.14 |
| 255 | A | autumn | October, 2016 | 2.73 | 1 | 227 | 3.85 |
| 256 | A | winter | December, 2017 | 5.3 | 2 | 271 | 4.15 |
| 257 | A | spring | April, 2017 | 6.4 | 2 | 315 | 4.15 |
| 258 | A | autumn | October, 2016 | 5.54 | 2 | 340 | 4.16 |
| 259 | A | autumn | October, 2016 | 4.79 | 2 | 353 | 4.18 |
| 260 | A | spring | May, 2017 | 7.21 | 2 | 324 | 4.18 |
| 261 | A | spring | April, 2017 | 6.4 | 2 | 334 | 4.19 |
| 262 | A | winter | December, 2017 | 6 | 2 | 296 | 4.19 |
| 263 | A | autumn | September, 2017 | 6.6 | 2 | 390 | 4.19 |
| 264 | A | spring | April, 2017 | 5.4 | 2 | 293 | 4.20 |
| 265 | A | spring | April, 2017 | 5.7 | 2 | 354 | 4.21 |
| 266 | A | autumn | October, 2016 | 4.94 | 2 | 359 | 4.21 |
| 267 | A | summer | August, 2017 | 6.02 | 2 | 298 | 4.21 |
| 268 | A | spring | May, 2017 | 7.8 | 2 | 294 | 4.22 |
| 269 | A | summer | August, 2017 | 6.15 | 2 | 295 | 4.23 |
| 270 | A | spring | April, 2017 | 6.4 | 2 | 341 | 4.25 |
| 271 | A | summer | August, 2017 | 5.65 | 2 | 403 | 4.25 |
| 272 | A | autumn | September, 2017 | 8.12 | 2 | 305 | 4.25 |
| 273 | A | winter | December, 2017 | 6 | 2 | 316 | 4.25 |
| 274 | A | spring | April, 2017 | 7.1 | 2 | 341 | 4.27 |
| 275 | A | autumn | September, 2017 | 7.1 | 2 | 379 | 4.27 |
| 276 | A | spring | May, 2017 | 7.07 | 2 | 287 | 4.28 |
| 277 | A | summer | August, 2017 | 7.1 | 2 | 248 | 4.28 |
| 278 | A | winter | January, 2017 | 4.81 | 2 | 360 | 4.29 |
| 279 | A | spring | April, 2017 | 5.4 | 2 | 358 | 4.30 |
| 280 | A | winter | January, 2017 | 6.73 | 2 | 323 | 4.30 |
| 281 | A | spring | April, 2017 | 6.8 | 2 | 349 | 4.30 |
| 282 | A | winter | December, 2017 | 5.92 | 2 | 298 | 4.32 |
| 283 | A | autumn | October, 2016 | 5.82 | 2 | 328 | 4.33 |
| 284 | A | autumn | October, 2016 | 5.41 | 2 | 300 | 4.35 |
| 285 | A | spring | April, 2017 | 6.9 | 2 | 390 | 4.38 |
| 286 | A | autumn | September, 2017 | 6.2 | 2 | 301 | 4.39 |
| 287 | A | winter | December, 2017 | 6.31 | 2 | 330 | 4.39 |
| 288 | A | winter | December, 2017 | 6 | 2 | 306 | 4.40 |
| 289 | B | spring | April, 2017 | 20.9 | 3 | 615 | 6.52 |
| 290 | C | winter | February,2017 | 27.6 | 5 | 640 | 7.23 |
| 291 | B | winter | December, 2017 | 15.57 | 4 | 724 | 5.71 |
| 292 | E | spring | April, 2017 | 16.8 | 4 | 764 | 5.93 |
| 293 | D | winter | February,2017 | 19.67 | 4 | 767 | 5.70 |
| 294 | C | winter | February,2017 | 30.48 | 5 | 645 | 7.75 |
| 295 | E | spring | April, 2017 | 16.1 | 4 | 785 | 7.16 |
| 296 | B | spring | April, 2017 | 28.19 | 6 | 646 | 7.31 |
| 297 | E | spring | April, 2017 | 18.66 | 5 | 647 | 6.50 |
| 298 | D | autumn | November, 2017 | 27.26 | 5 | 647 | 7.13 |
| 299 | B | spring | April, 2017 | 26.26 | 6 | 653 | 6.76 |
| 300 | E | spring | April, 2017 | 16.1 | 4 | 787 | 5.75 |
| 301 | B | autumn | November, 2017 | 19.14 | 5 | 668 | 6.08 |
| 302 | C | winter | February,2017 | 24.52 | 5 | 674 | 6.82 |
| 303 | E | winter | December, 2017 | 18.98 | 5 | 677 | 6.83 |
| 304 | A | summer | August, 2017 | 6.27 | 2 | 342 | 4.42 |
| 305 | A | autumn | September, 2017 | 7.3 | 2 | 365 | 4.42 |
| 306 | A | winter | December, 2017 | 4.3 | 1 | 229 | 3.76 |
| 307 | A | winter | January, 2017 | 2.6 | 1 | 230 | 3.38 |
| 308 | A | spring | April, 2017 | 6.3 | 2 | 298 | 4.42 |
| 309 | A | autumn | September, 2017 | 7.2 | 2 | 377 | 4.43 |
| 310 | A | autumn | September, 2017 | 3.57 | 1 | 231 | 3.50 |
| 311 | A | autumn | October, 2016 | 5.42 | 2 | 303 | 4.43 |
| 312 | A | winter | December, 2017 | 4.1 | 1 | 232 | 3.64 |
| 313 | A | winter | December, 2017 | 3.83 | 1 | 233 | 3.64 |
| 314 | A | autumn | September, 2017 | 6.34 | 2 | 379 | 4.44 |
| 315 | A | autumn | September, 2017 | 3.16 | 1 | 233 | 3.32 |
| 316 | A | spring | April, 2017 | 8.4 | 2 | 384 | 4.46 |
| 317 | A | autumn | September, 2017 | 3.16 | 1 | 234 | 3.37 |
| 318 | A | autumn | October, 2016 | 6.57 | 2 | 355 | 4.47 |
| 319 | A | autumn | September, 2017 | 4.08 | 1 | 235 | 3.56 |
| 320 | A | winter | December, 2017 | 3.93 | 1 | 235 | 3.54 |
| 321 | A | winter | January, 2017 | 3.01 | 1 | 235 | 3.70 |
| 322 | A | winter | December, 2017 | 4.7 | 1 | 236 | 3.71 |
| 323 | A | winter | December, 2017 | 3.6 | 1 | 237 | 3.45 |
| 324 | A | winter | December, 2017 | 5.8 | 1 | 238 | 3.49 |
| 325 | A | autumn | September, 2017 | 4.65 | 1 | 240 | 3.50 |
| 326 | A | autumn | September, 2017 | 6.4 | 2 | 398 | 4.49 |
| 327 | A | autumn | September, 2017 | 3.79 | 1 | 240 | 3.46 |
| 328 | A | autumn | September, 2017 | 3.63 | 1 | 240 | 3.40 |
| 329 | A | summer | August, 2017 | 6.78 | 2 | 342 | 4.50 |
| 330 | A | winter | January, 2017 | 3.24 | 1 | 240 | 3.61 |
| 331 | A | autumn | September, 2017 | 7.23 | 2 | 395 | 4.50 |
| 332 | A | spring | April, 2017 | 4.35 | 1 | 241 | 3.71 |
| 333 | A | spring | May, 2017 | 3.91 | 1 | 241 | 4.18 |
| 334 | A | spring | May, 2017 | 2.71 | 1 | 241 | 3.61 |
| 335 | D | winter | February,2017 | 22.45 | 5 | 679 | 6.49 |
| 336 | A | winter | December, 2017 | 4.33 | 1 | 243 | 3.66 |
| 337 | A | autumn | September, 2017 | 9.43 | 2 | 415 | 4.50 |
| 338 | A | autumn | October, 2016 | 2.1 | 1 | 243 | 3.43 |
| 339 | B | spring | April, 2017 | 20.93 | 6 | 682 | 6.41 |
| 340 | A | winter | January, 2017 | 6.02 | 2 | 347 | 4.50 |
| 341 | E | spring | April, 2017 | 17.31 | 5 | 685 | 6.55 |
| 342 | A | autumn | September, 2017 | 8.2 | 2 | 354 | 4.51 |
| 343 | A | winter | December, 2017 | 3.7 | 1 | 246 | 3.48 |
| 344 | A | autumn | September, 2017 | 7.4 | 2 | 424 | 4.51 |
| 345 | A | spring | April, 2017 | 8 | 2 | 379 | 4.53 |
| 346 | A | autumn | September, 2017 | 7.53 | 2 | 370 | 4.54 |
| 347 | A | autumn | September, 2017 | 7.18 | 2 | 335 | 4.54 |
| 348 | A | autumn | September, 2017 | 7.05 | 2 | 346 | 4.55 |
| 349 | A | spring | May, 2017 | 4.2 | 1 | 247 | 3.21 |
| 350 | A | winter | December, 2017 | 4.6 | 1 | 248 | 3.73 |
| 351 | A | winter | January, 2017 | 6.26 | 2 | 398 | 4.55 |
| 352 | A | spring | May, 2017 | 7.63 | 2 | 355 | 4.55 |
| 353 | A | spring | May, 2017 | 8.83 | 2 | 342 | 4.56 |
| 354 | A | winter | December, 2017 | 4.26 | 1 | 250 | 3.59 |
| 355 | A | winter | December, 2017 | 4.22 | 1 | 251 | 3.71 |
| 356 | A | winter | December, 2017 | 4.82 | 1 | 252 | 3.91 |
| 357 | A | spring | May, 2017 | 4.71 | 1 | 252 | 3.89 |
| 358 | A | winter | January, 2017 | 3.03 | 1 | 254 | 3.60 |
| 359 | A | winter | December, 2017 | 4.9 | 1 | 255 | 3.61 |
| 360 | A | winter | December, 2017 | 4.2 | 1 | 256 | 3.57 |
| 361 | A | autumn | September, 2017 | 7.48 | 2 | 368 | 4.56 |
| 362 | B | winter | December, 2017 | 7.08 | 2 | 385 | 4.57 |
| 363 | A | autumn | September, 2017 | 7.48 | 2 | 309 | 4.58 |
| 364 | A | winter | January, 2017 | 3.51 | 1 | 256 | 3.87 |
| 365 | A | autumn | September, 2017 | 4.1 | 1 | 258 | 3.70 |
| 366 | A | winter | December, 2017 | 3.86 | 1 | 262 | 3.60 |
| 367 | A | summer | August, 2017 | 4.81 | 1 | 265 | 4.12 |
| 368 | A | summer | August, 2017 | 4.89 | 1 | 270 | 3.98 |
| 369 | A | autumn | September, 2017 | 4.35 | 1 | 273 | 3.91 |
| 370 | A | winter | December, 2017 | 5.1 | 1 | 278 | 4.04 |
| 371 | A | spring | May, 2017 | 5.53 | 1 | 279 | 4.02 |
| 372 | A | spring | April, 2017 | 4.3 | 1 | 282 | 4.06 |
| 373 | A | spring | May, 2017 | 5.45 | 1 | 283 | 4.11 |
| 374 | A | summer | August, 2017 | 6.85 | 2 | 345 | 4.61 |
| 375 | A | winter | December, 2017 | 4.89 | 1 | 286 | 3.81 |
| 376 | A | winter | December, 2017 | 5.25 | 1 | 294 | 4.10 |
| 377 | A | spring | April, 2017 | 7.7 | 2 | 379 | 4.62 |
| 378 | E | spring | April, 2017 | 18.9 | 5 | 687 | 6.14 |
| 379 | A | autumn | September, 2017 | 8.6 | 2 | 409 | 4.65 |
| 380 | A | winter | December, 2017 | 8.17 | 2 | 305 | 4.66 |
| 381 | A | spring | May, 2017 | 8.24 | 2 | 360 | 4.66 |
| 382 | A | summer | August, 2017 | 5.96 | 1 | 295 | 2.72 |
| 383 | A | autumn | September, 2017 | 8.17 | 2 | 345 | 4.71 |
| 384 | A | spring | May, 2017 | 9.05 | 2 | 364 | 4.72 |
| 385 | A | spring | May, 2017 | 9.83 | 2 | 334 | 4.76 |
| 386 | A | winter | December, 2017 | 7.95 | 2 | 329 | 4.82 |
| 387 | A | autumn | September, 2017 | 7.6 | 2 | 376 | 4.83 |
| 388 | E | spring | April, 2017 | 31.8 | 5 | 687 | 7.79 |
| 389 | A | summer | August, 2017 | 7.58 | 2 | 375 | 4.84 |
| 390 | B | autumn | November, 2017 | 17.7 | 5 | 688 | 6.08 |
| 391 | E | spring | April, 2017 | 17.33 | 5 | 689 | 6.27 |
| 392 | A | spring | May, 2017 | 10.23 | 2 | 336 | 4.86 |
| 393 | B | winter | December, 2017 | 8.99 | 2 | 396 | 4.89 |
| 394 | A | summer | August, 2017 | 5.46 | 1 | 296 | 3.54 |
| 395 | A | summer | August, 2017 | 8.51 | 2 | 333 | 4.95 |
| 396 | E | spring | April, 2017 | 18.6 | 5 | 705 | 6.58 |
| 397 | A | autumn | September, 2017 | 6.6 | 2 | 361 | 5.03 |
| 398 | E | spring | April, 2017 | 29.77 | 6 | 711 | 6.67 |
| 399 | A | autumn | September, 2017 | 8.41 | 2 | 395 | 5.11 |
| 400 | D | winter | November,2017 | 24.83 | 6 | 712 | 6.97 |
| 401 | E | spring | April, 2017 | 27.09 | 6 | 712 | 6.56 |
| 402 | E | spring | April, 2017 | 28.33 | 5 | 724 | 7.72 |
| 403 | E | spring | April, 2017 | 27 | 5 | 738 | 6.69 |
| 404 | C | winter | February,2017 | 29.31 | 5 | 750 | 7.44 |
| 405 | E | spring | April, 2017 | 28.23 | 5 | 751 | 7.75 |
| 406 | D | winter | February,2017 | 26.07 | 5 | 753 | 6.84 |
| 407 | B | spring | April, 2017 | 25.46 | 6 | 756 | 6.87 |
| 408 | C | winter | February,2017 | 31.22 | 5 | 770 | 7.56 |
| 409 | C | winter | February,2017 | 27.38 | 5 | 795 | 7.41 |
